# The Effect of MicroRNA-126 Mimic Administration on Vascular Perfusion Recovery in an Animal Model of Hind Limb Ischemia

**DOI:** 10.3389/fmolb.2021.724465

**Published:** 2021-08-25

**Authors:** Panagiotis Theofilis, Georgia Vogiatzi, Evangelos Oikonomou, Maria Gazouli, Gerasimos Siasos, Hector Katifelis, Despoina Perrea, Manolis Vavuranakis, Dimitrios C Iliopoulos, Costas Tsioufis, Dimitris Tousoulis

**Affiliations:** ^1^ First Department of Cardiology, “Hippokration” General Hospital, University of Athens Medical School, Athens, Greece; ^2^ Third Department of Cardiology, Thoracic Diseases General Hospital “Sotiria”, University of Athens Medical School, Athens, Greece; ^3^ Department of Basic Medical Sciences, Laboratory of Biology, National and Kapodistrian University of Athens, Athens, Greece; ^4^ Laboratory of Experimental Surgery and Surgical Research “N.S. Christeas”, University of Athens Medical School, Athens, Greece

**Keywords:** peripheral arterial disease, angiogenesis, VEGF, MicroRNA-126, limb ischemia

## Abstract

**Background:** MicroRNAs have been linked to angiogenesis and could prove to be valuable future therapeutic targets in ischemic cardiovascular diseases.

**Methods:** Ten-week-old male C57Bl/6 mice were subjected to left femoral artery ligation and were treated with microRNA-126 mimic at a dose of 5 mg/kg (Group A, *n* = 10) or 5 mg/kg microRNA mimic negative control (Group B, *n* = 10) on days 1, 3, and 7. Laser Doppler imaging was performed to verify successful ligation on day 0 and to evaluate differences in the ischemic-to-normal (I/N) hind limb perfusion ratio on day 28. Muscle tissue expression of microRNA-126 and vascular endothelial growth factor (VEGF) was determined *via* PCR.

**Results:** Following microRNA-126 mimic administration in Group A subjects, we noted a stepwise increase in I/N hind limb perfusion ratio (Day 0: 0.364 ± 0.032 vs. Day 8: 0.788 ± 0.049 vs. Day 28: 0.750 ± 0.039, *p* = 0.001). In Group B a stepwise increase in I/N hind limb perfusion ratio was observed (Day 0: 0.272 ± 0.057 vs. Day 8: 0.382 ± 0.020 vs. Day 28: 0.542 ± 0.028, *p* = 0.074). Muscle tissue expression of microRNA-126 in the ischemic hind limb of Group A was 350-fold lower compared to the ischemic hind limb of Group B (*p* < 0.001). A higher expression (14.2-fold) of VEGF in the ischemic hind limb of microRNA-126-treated mice compared to that of control group was detected (*p* < 0.001). A statistically significant negative correlation was noted between microRNA-126 and VEGF tissue expression levels in the ischemic limbs of the entire study population.

**Conclusion:** MicroRNA-126 delivery in the ischemic hind limb of mice improved vascular perfusion with VEGF upregulation.

## Introduction

Peripheral arterial disease (PAD) caused mainly by atherosclerosis portent significant morbidity, adverse prognosis and even mortality (Aboyans et al., 2018). Critical limb ischemia is considered the most aggressive form of PAD due to the lower survival rates noted in the subgroup even when compared to heart failure, stroke and various cancer types (Mustapha et al., 2018). Localized treatment approach aims to alleviate symptoms, improve circulation and to address risk related to a specific lesion.

Recently, scientific interest has been shifted towards epigenomics, and especially non coding RNA-based therapeutic and diagnostic approaches in cardiovascular medicine, including microRNAs (Lu and Thum, 2019; Zheng et al., 2020), since their dysregulated expression has been noted in cardiovascular diseases (Vogiatzi et al., 2018; Siasos et al., 2020). Diagnostically they have been tested in various cardiovascular diseases, namely coronary artery disease, PAD and heart failure (Siasos et al., 2014; Economou et al., 2015; Vogiatzi et al., 2018). Moreover, their role in ischemia/reperfusion injury has also been described, possibly by the regulation of oxidative stress-promoting pathways (Carbonell and Gomes, 2020). Their therapeutic potential has already been a topic of intense scientific research especially in oncology, since changes in their expression have been linked with tumorigenesis and their targeting could lead to successful management of various cancer types (McManus, 2003; Cho, 2010).

In the context of atherosclerosis, several microRNAs have been linked to angiogenesis and could prove to be valuable future therapeutic targets (van Balkom et al., 2013; Beltrami et al., 2017; Ma et al., 2018). MicroRNA-126 is believed to influence angiogenesis *via* interaction with the vascular endothelial growth factor (VEGF)/phosphoinositide 3-kinase (PI3K)/Akt pathway, highlighting its emerging role in oncology (Ghafouri-Fard et al., 2021). Based on those findings, we investigated the speculated pro-angiogenic effect of microRNA-126 through administration of a mimic analogue in an *in vivo* model of hind limb ischemia, attempting to improve the knowledge of the underlying molecular pathways and demonstrate its potential *in vivo* effects.

## Materials and Methods

### Animal Experiments

Ten-week-old adult male C57Bl/6 mice (*n* = 20) weighing 23–28 g were obtained from the National Center for Scientific Research “Demokritos” (Athens, Greece) and were given free access to food and water. Animals were anesthetized using a combination of ketamine and xylazine in doses of 100 mg/kg and 10 mg/kg respectively, followed by femoral artery ligation of the left hind limb *via* a 6–0 nylon suture in order to create critical limb ischemia, while the right hind limb was left intact. After the surgical procedure (day 0), mice were randomly divided into two groups. Group A mice (*n* = 10) were treated with intramuscular administration of a microRNA-126 mimic (ThermoFisher Scientific, USA) according to an ad-hoc protocol on days 1, 3 and 7 at a dose of 5 mg/kg, according to previous experience (Desjarlais et al., 2017). Group B mice (i.e., control group, *n* = 10) received intramuscular injections of 5 mg/kg microRNA negative control at the same time points. On day 28, mice were euthanized by prolonged ether inhalation with harvested muscle tissue specimens being deep frozen in liquid nitrogen and then stored at −80°C (Supplementary Figure S1).

The protocol was approved by the ethics committee of the National and Kapodistrian University of Athens Medical School and was carried out in accordance to the national law for laboratory animals’ protection, with all efforts being made to minimize animal suffering.

### Laser Doppler Perfusion Imaging

Following the surgical ligation of femoral artery, scanning with a laser doppler flowmeter (Perimed, Sweden) was applied in order to verify the successful occlusion after brief anesthesia using ether inhalation. After stabilization of the mouse under the flowmeter, three consecutive scans of 3 min duration were performed, with regions of interest being drawn across the hind limbs. The mean perfusion of ischemic and non-ischemic hind limbs after the three measurements was used and the data are expressed as the ratio of mean perfusion in the ischemic hind limb to the mean perfusion in the non-ischemic hind limb (I/N). Scanning was repeated on day 8 and prior to euthanasia (Day 28) to assess alterations in perfusion according to group.

### Quantitative Real-Time PCR

Muscle tissue was harvested from the quadriceps femoris muscle of the ischemic and the non-ischemic hind limb of mice. According to our protocol, six mice from each group were randomly selected for the real-time PCR (rtPCR) analysis, prior to the induction of hind limb ischemia. Total RNA was extracted from each sample using Trizol (Invitogen, ΤRI Reagent) according to the manufacturer’s instructions RNA was reverse transcribed using gene specific looped primers (Rio et al., 2010) by revert aid cDNA synthesis kit (Thermo Fisher Scientific, Waltham, Massachusetts, USA). RtPCR) was performed to assess the mRNA levels of microRNA-126 and vascular endothelial growth factor (VEGF). Real time PCR was performed using ABI PRISM 7500 Real time PCR system (Applied Biosystems). The parameters followed were: Initial denaturation—94°C for 2 min, denaturation- 94°C for 30 s, annealing- 60°c for 1 min for 40 cycles. 20 μl reaction containing 7 μl MQ, 10 μl SYBRR Green universal PCR Master Mix (Applied Biosystems, California, USA), 1 μl of each forward and reverse primer (4 picomole/μl), 1 μl cDNA. The relative expression level of microRNA-126 was normalized to U6 small nuclear RNA expression, and mRNA expression was normalized to the housekeeping gene GAPDH *via* the 2^−ΔΔCT^ method (Livak and Schmittgen, 2001). The primer sequences used in our study are listed in Supplementary Table S1.

### Statistical Analysis

Data are expressed as mean ± standard error of mean. Between group differences were tested *via* Mann-Whitney U test. Repeated measures analysis of variance (Friedman’s ANOVA) was performed to explore the differences in limb perfusion over time. Correction for multiple comparisons was applied when indicated. A general linear model was applied to test for interaction of follow-up limb perfusion changes according to intervention. Correlation coefficient between rtPCR results was assessed using the Spearman correlation coefficient. All reported *p* values were based on two-sided hypotheses. A *p* value of <0.05 was considered statistically significant. All statistical calculations were performed using SPSS software (version 25.0; SPSS Inc., Chicago, Illinois, USA).

## Results

### MicroRNA-126 Mimic Administration and Vascular Perfusion in the Ischemic Hind Limb

**FIGURE 1:**
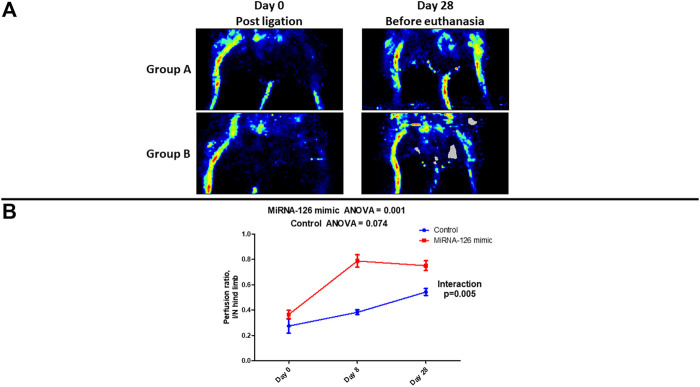
**(A)** Visual evaluation of laser doppler perfusion imaging examination showing the changes in vascular perfusion of ischemic hind limb after surgical ligation of femoral artery and on the 28th day, following treatment with microRNA-126 mimic (Group A) or microRNA mimic negative control (Group B). **(B)** Quantitative assessment of changes in the ischemic/normal hind limb perfusion ratio *via* laser doppler examination showing a higher stepwise improvement in microRNA-126 mimic treated mice, as noted by a statistically significant *p*-value for the interaction between study group and ischemic/normal hind limb perfusion ratio.

Visual evaluation of laser doppler perfusion imaging confirmed that in the entire study population (Groups A and B) ligation of the left femoral artery resulted in significant perfusion defect. (Figure 1A). Visual reevaluation with laser doppler perfusion imaging at the 28th day revealed restoration of perfusion in the ischemic hind limb of Group A (microRNA-126-treated group) while there was significant perfusion defect in the ischemic hind limb of Group B (control group) (Figure 1A).

Post-ligation (Day 0) quantitative (laser doppler) assessment of limb perfusion demonstrated similar I/N hind limb perfusion ratio in the two study groups (Group A: 0.364 ± 0.032 vs. Group B: 0.272 ± 0.057, *p* = 0.15). Repeated measures ANOVA revealed that there was a stepwise increase in I/N hind limb perfusion ratio in the Group A (Day 0: 0.364 ± 0.032 vs. Day 8: 0.788 ± 0.049 vs. Day 28: 0.750 ± 0.039, *p* = 0.001). In the Group B a stepwise increase in I/N hind limb perfusion ratio was also observed (Day 0: 0.272 ± 0.057 vs. Day 8: 0.382 ± 0.020 vs. Day 28: 0.542 ± 0.028, *p* = 0.074). Interestingly, in group A, even as early as day 8th from ligation, a significant improvement in I/N compared to Day 0 was observed (Table 1).

**TABLE 1:** Laser doppler examination of ischemic/normal hind limb perfusion ratio on Day 0, Day 8 and Day 28 according to treatment group.

	Day 0	Day 8	Day 28	p
Group A	0.364 ± 0.032	0.788 ± 0.049^a^ ^b^	0.750 ± 0.039^a^ ^b^	0.001
Group B	0.272 ± 0.057	0.382 ± 0.020	0.542 ± 0.028	0.074

Data presented as mean ± standard error of mean.

a Indicates *p* < 0.01 when compared to Day 0.

b Indicates *p* < 0.01 when compared to group B in the equivalent time point.

Importantly, over time changes of I/N hind limb perfusion ratio were significantly higher in Group A (treatment group) compared to Group B (control group) (p for interaction = 0.005) (Figure 1B) (Table 1).

### Effects of microRNA-126 Mimic Administration on Tissue Expression of microRNA-126 and Angiogenetic Factors.

**FIGURE 2:**
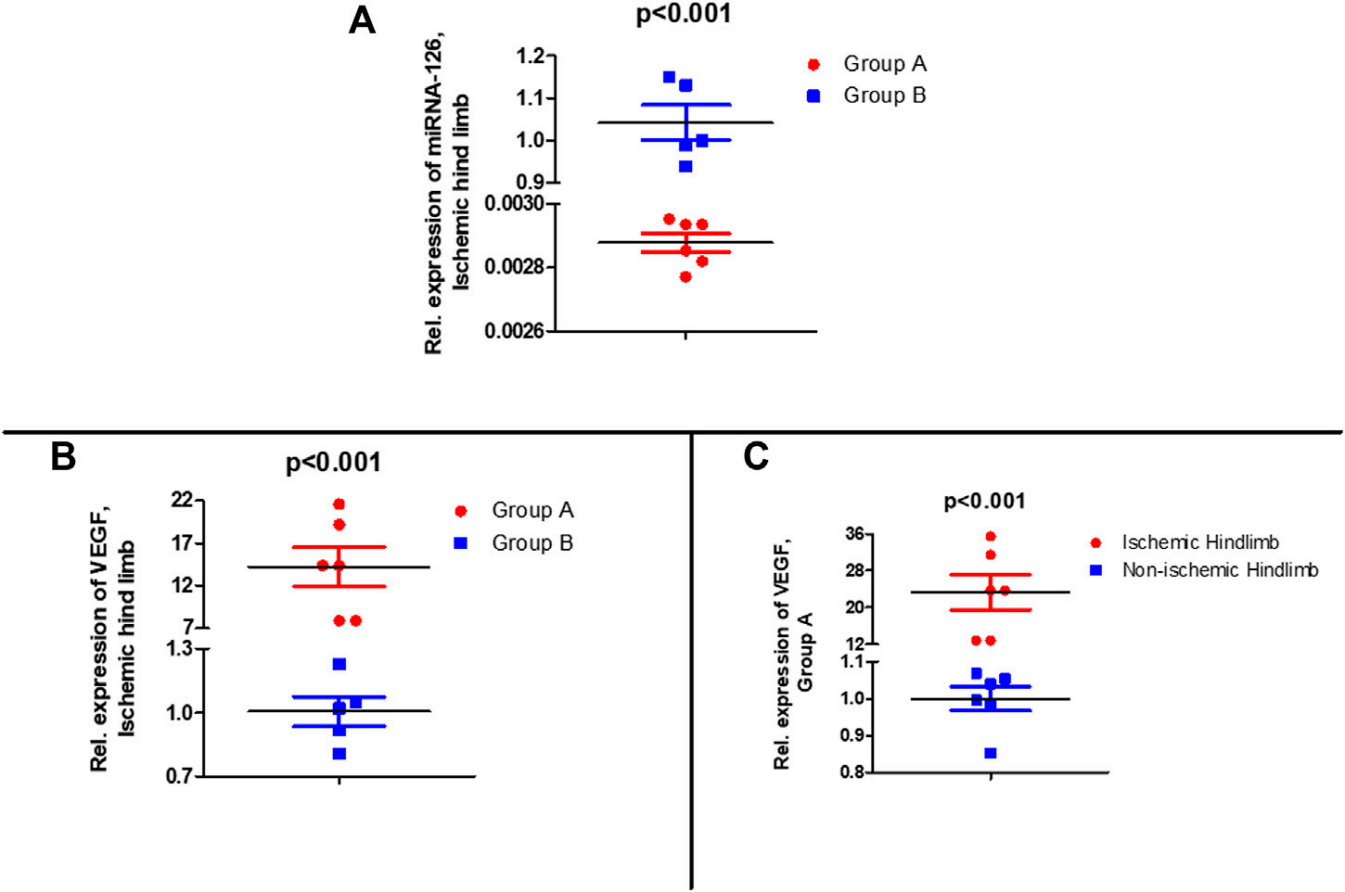
**(Α)** Relative expression of microRNA-126 in muscle tissue of the ischemic hind limb of microRNA-126 treated group compared control group after euthanasia (28th day), **(B)** Relative expression of microRNA-126 vascular endothelial growth factor (VEGF) in muscle tissue of the ischemic hind limb of microRNA-126 treated group compared control group after euthanasia (28th day), **(C)** Relative expression of VEGF in muscle tissue of the ischemic hind limb compared to non-ischemic hind limb of microRNA-126 treated group after euthanasia (28th day).

3 weeks (time of euthanasia) from the last administration of experimental drug (microRNA-126 mimic or microRNA mimic negative control), in the microRNA-126 mimic treated group (Group A), muscle tissue expression of microRNA-126 in the ischemic hind limb was 350-fold lower compared to the ischemic hind limb of Group B (control group) (*p* < 0.001) (Figure 2A).

At the same time point (time of euthanasia) tissue expression of VEGF was measured to examine its potential effect in restoration of perfusion in ischemic hind limb and how administration of microRNA-126 mimic may affect its expression. A higher expression (14.2-fold) of VEGF in the ischemic hind limb of microRNA-126-treated mice (Group A) compared to that of control group (Group B) (*p* < 0.001) (Figure 2B) was observed. In group A there was higher expression (23.4-fold) of VEGF in the ischemic hind limb compared to non-ischemic hind limb (*p* < 0.001) (Figure 2C).

**FIGURE 3:**
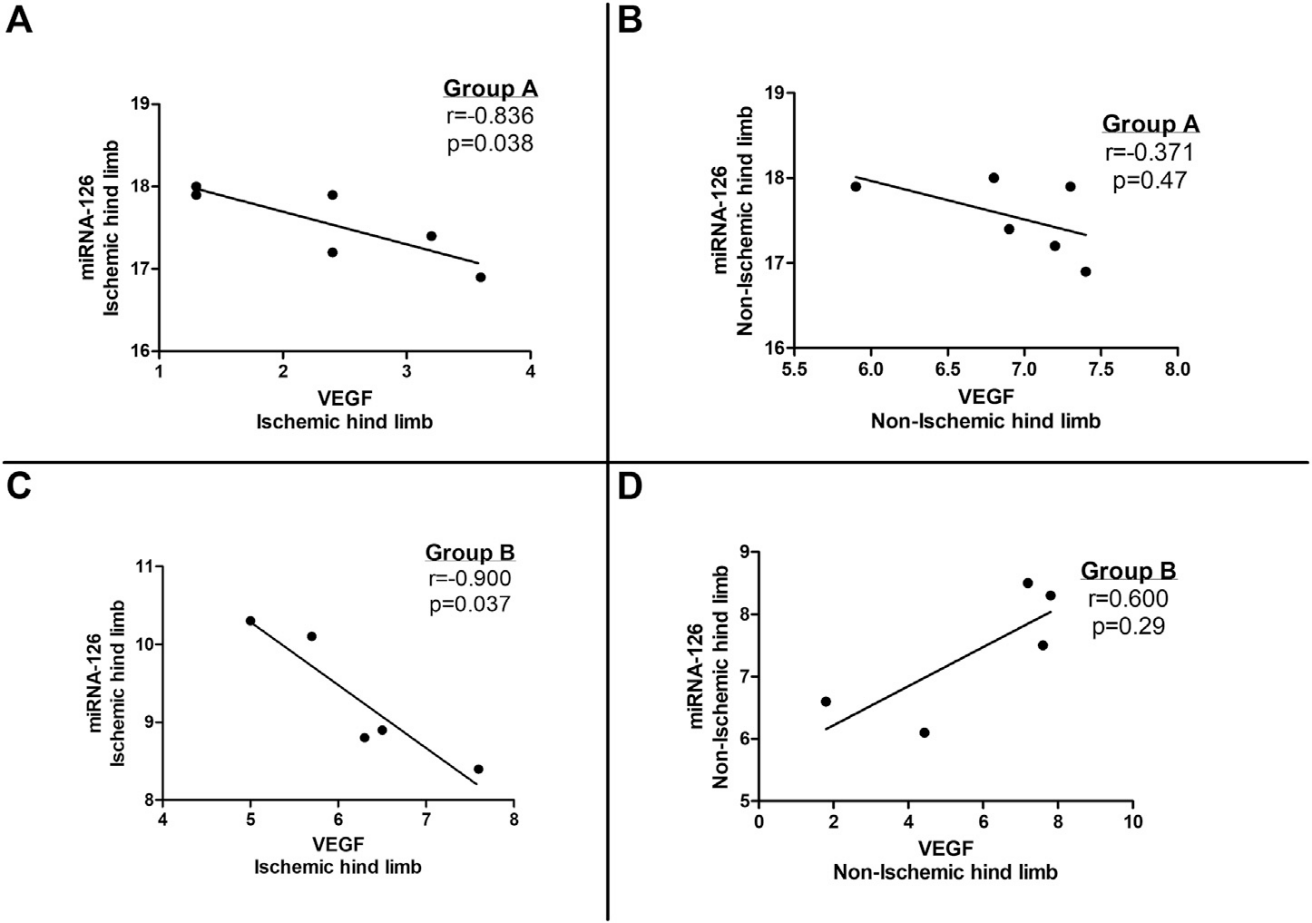
Correlation between tissue vascular endothelial growth factor (VEGF) and microRNA-126 expression in **(A)** the ischemic hind limb and **(B)** the non-ischemic hind limb of microRNA-126 treated mice (Group A) as well as in **(C)** the ischemic hind limb and **(D)** the non-ischemic hind limb of the control group (Group B).

A statistically significant negative correlation was noted between microRNA-126 and VEGF tissue expression levels in the ischemic limbs of both group A and B subjects (Figures 3A, C) whereas no correlation was observed between microRNA-126 and VEGF levels in the non-ischemic limbs of the entire study population (groups A and B) (Figures 3B, D).

## Discussion

In our study, we showed that administration of microRNA-126 mimic in hind limb ischemic mice improves vascular perfusion even at the early post administration period a result that was evident also as late as 28 days following femoral artery ligation. Moreover, we found that improved perfusion post femoral artery ligation and microRNA-126 mimic administration was associated with increased VEGF tissue expression levels. Interestingly, in the late post administration phase, tissue expression levels of microRNA-126 were minimal in opposite to the increased VEGF levels and achieved hind limb perfusion.

Angiogenesis, a physiologic process aiming at vessel regeneration in the context of tissue damage or hypoxia, is a complex procedure consisting of enzymatic breaking of capillary basement membrane, multiplication and migration of endothelial cells, differentiation and specialization (Gerhardt, 2008). Mechanisms of hypoxia detection are crucial for initiation of the angiogenesis cascade while the hypoxia-induced VEGF release is an important mediator (Gerhardt, 2008). Therapy involving growth factors in PAD aiming at tissue regeneration and angiogenesis with VEGF and hepatocyte growth factor administration, has been applied in the past with encouraging results (Barc et al., 2020; Reijven and Soeters, 2020).

MicroRNAs, as an epigenomic mechanism, pose an attractive approach in the induction of angiogenesis and have been used *in vitro* and *in vivo* (Kesidou et al., 2020). Since angiogenesis is a process involving numerous genes (Fröhlich, 2019), repression of several angiogenesis inhibitors through microRNA treatment has resulted in more efficient vascular regeneration through upregulated expression of growth factors (VEGF, basic fibroblast growth factor) and transcription-facilitation molecules (hypoxia-inducible factor-1) (Dentelli et al., 2010; Rey and Semenza, 2010). Indeed, the pro-angiogenic microRNA-93 promoted angiogenesis on a mouse model of hind limb ischemia through inhibition of Cyclin-dependent kinase inhibitor 1A, which has been linked with endothelial dysfunction and is implicated in the development of PAD (Kesidou et al., 2020). Furthermore, microRNA-92a when overexpressed in endothelial cells blocked angiogenesis. When microRNA-92a antagomir was administered in mice with hind limb ischemia, functional recovery of the damaged tissue was observed through upregulation of proangiogenic proteins and integrin subunit alpha5 (Bonauer et al., 2009).

In our mouse model of hind limb ischemia, we documented recovery of perfusion in the microRNA-126 mimic treated group. MicroRNA-126 may regulate the endothelial cells’ response to VEGF by depressing negative regulators of VEGF pathway including the Sprouty-related protein SPRED1 and phosphoinositol-3 kinase regulatory subunit 2 (PIK3R2/p85-beta) (Fish et al., 2008). Indeed, we documented increase tissue levels of VEGF expression in the ischemic limb of microRNA-126 mimic treated mice. Enhanced microRNA-126 activity may have preceded regenerative mechanisms including vessel remodeling, angiogenesis and neurogenesis (Rey and Semenza, 2010), while inactivation of PIK3R2 by microRNA-126 accelerates skin wound healing and angiogenesis after treatment with exosomes expressing microRNA-126, as reported in the study of Zhang et al. (2020).

Interestingly, when tissue expression of microRNA-126 was measured post euthanasia (21 days from the last administration of microRNA mimic) we documented a significant downregulation of this microRNA expression in the microRNA-126 mimic treated animals compared to controls. The limited life of exogenously administered microRNA-126 mimic may partially explain this observation. Moreover, the prolonged ischemia in the control group may have acted as a stimulus for continuous endogenous expression of microRNA-126. On the contrary, the quicker restoration of perfusion in the microRNA-126-treated group may have resulted in minimal endogenous expression of microRNA-126. Importantly, Cao et al. attempted ultrasound-mediated delivery of microRNA-126 in a similar experimental model (Cao et al., 2015). Even though they detected significantly elevated microRNA-126 levels in the microRNA-126-treated mice compared to the control group 3 days after delivery, no significant differences were noted between the study groups on the 14th day following the intervention, while an improvement in ischemic hind limb perfusion was also observed in microRNA-126-treated mice, in line with our findings (Cao et al., 2015). To further understand our findings, we documented an inverse association between VEGF tissue expression and microRNA-126 levels which may possibly imply a negative feedback regulation of the microRNA-126/VEGF signaling pathway, highlighting the complexity of the epigenomic mechanisms involved in angiogenesis.

Our study has several limitations. To begin with, angiogenesis was not examined through the measurement of capillary density per unit area which, together with the over-time changes in endothelial cell proliferation following microRNA-126 mimic treatment, could provide further insight and will be performed in future studies. Moreover, the effect of microRNA-126 mimic on vascular remodeling and arteriogenesis has not been assessed in the current study and will be examined in the future. Furthermore, proangiogenic cells, which are known contributors in the interplay of VEGF with angiogenesis, could not be evaluated based on the present experimental design. Lastly, western blotting was not utilized to evaluate VEGF protein expression as well as downstream molecules of the VEGF signaling pathway (SPRED-1, PIK3R2, protein kinase B) and will be performed in upcoming studies.

## Conclusion

MicroRNA-126 delivery in the ischemic hind limb of mice can improve vascular perfusion *via* angiogenesis, which is mediated by VEGF expression. Based on the inverse association of VEGF expression and tissue levels of microRNA-126 a negative feedback regulation of the microRNA-126/VEGF signaling pathway is suggestive. Whether microRNA modulation of vascular regeneration in PAD *via* induction of angiogenesis could end up being a future therapeutic target in ischemic cardiovascular diseases merits further research.

## Data Availability

The raw data supporting the conclusion of this article will be made available by the authors, without undue reservation.
