# Supplementary material for: The Effect of MicroRNA-126 Mimic Administration on Vascular Perfusion Recovery in an Animal Model of Hind Limb Ischemia
Source: Front Mol Biosci. 2021 Aug 25;8:724465. doi: 10.3389/fmolb.2021.724465 (PMC8423909; doi:10.3389/fmolb.2021.724465)
Supplement: Supplementary file 1 [file Table1.DOCX]

| **Supplementary Table 1: Sequences of real-time PCR primers used in the study** | |
| --- | --- |
| **Name** | **Sequence (5΄-3΄)** |
| **Mir-126** | F: 5’-GCAATTGCACTGGA-TACGACCGCATTA-3’  R: 5’-CAGTGCGTGTCGTGGAGT-3 |
| **VEGF** | F: 5’-TGTACCTCCACCATGCCAAGT-3’  R: 5’-TGGTAGACGTCCATGAACTTG-3’ |
| **U6sn** | F: 5’-CTCGCTTCGGCAGCACA-3’  R: 5’-AACGCTTCACGAATTTGCGT-3’ |
| **GAPDH** | F: 5’-CGACTTCAACAGCAACTCCCACTCTTCC-3’  R: 5’-TGGGTGGTCCAGGGTTTCTTACTCCTT-3’ |
