# Supplementary figures and images for: The Effect of MicroRNA-126 Mimic Administration on Vascular Perfusion Recovery in an Animal Model of Hind Limb Ischemia
Source: Front Mol Biosci. 2021 Aug 25;8:724465. doi: 10.3389/fmolb.2021.724465 (PMC8423909; doi:10.3389/fmolb.2021.724465)

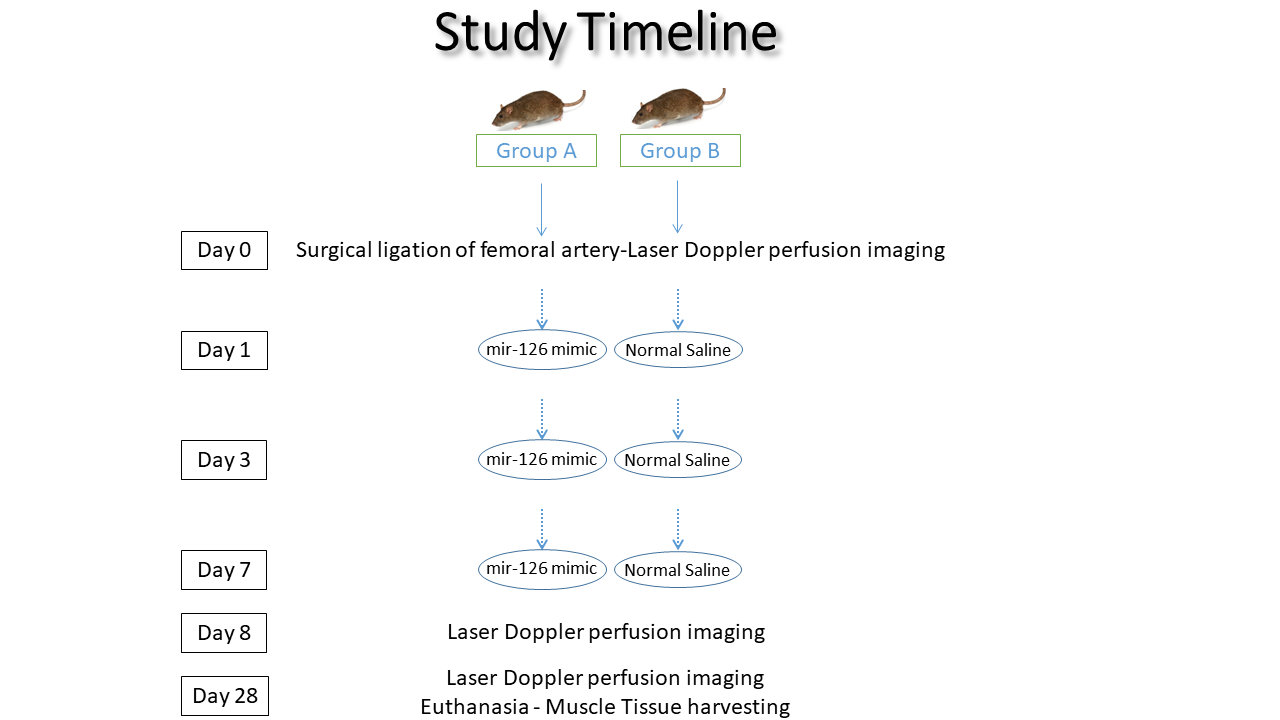

Supplement: Supplementary file 2 [file Image1.TIF]
